# Description of a new species of Tardigrada *Hypsibius* *nivalis* sp. nov. and new phylogenetic line in Hypsibiidae from snow ecosystem in Japan

**DOI:** 10.1038/s41598-022-19183-8

**Published:** 2022-09-02

**Authors:** Masato Ono, Nozomu Takeuchi, Krzysztof Zawierucha

**Affiliations:** 1 Graduate School of Science and Engineering, Chiba University, Chiba, Japan; 2 Department of Earth Sciences, Graduate School of Science, Chiba University, Chiba, Japan; 3 Department of Animal Taxonomy and Ecology, Adam Mickiewicz University, Poznań, Poland; 4 Department of Biological and Environmental Science, University of Jyväskylä, Jyväskylä, Finland; 5 Faculty of Forestry and Wood Sciences, Czech University of Life Sciences, Prague, Czech Republic

**Keywords:** Zoology, Entomology

## Abstract

Snow ecosystems are an important component of polar and mountainous regions, influencing water regime, biogeochemical cycles and supporting snow specific taxa. Although snow is considered to be one of the most unique, and at the same time a disappearing habitat, knowledge of its taxonomic diversity is still limited. It is true especially for micrometazoans appearing in snow algae blooming areas. In this study, we used morphological and molecular approaches to identify two tardigrade species found in green snow patches of Mt. Gassan in Japan. By morphology, light (PCM) and scanning electron microscopy (SEM), and morphometry we described *Hypsibius* *nivalis* sp. nov. which differs from other similar species by granular, polygonal sculpture on the dorsal cuticle and by the presence of cuticular bars next to the internal claws. Additionally, phylogenetic multilocus (COI, 18S rRNA, 28S rRNA) analysis of the second taxon, *Hypsibius* sp. identified by morphology as *convergens-pallidus* group, showed its affinity to the Hypsibiidae family and it is placed as a sister clade to all species in the Hypsibiinae subfamily. Our study shows that microinvertebrates associated with snow are poorly known and the assumption that snow might be inhabited by snow-requiring tardigrade taxa cannot be ruled out. Furthermore, our study contributes to the understanding subfamily Hypsibiinae showing that on its own the morphology of specimens belonging to *convergens*-*pallidus* group is insufficient in establishing a true systematic position of specimens.

## Introduction

Seasonal snow in mountains is an ephemeral cold environment that melts completely by end of the summer^1^. Despite harsh conditions such as low temperature and high UV irradiation, many organisms are well-adapted to the snow environment, and they are represented by primary producers (snow algae and cyanobacteria), microbial heterotrophs (ciliates and fungi), and consumers (invertebrates)^2–5^. Numerous attempts have been made to recognize and describe the snow ecosystems, but snow’s biodiversity has still been poorly recognised, the best examples of which are minute invertebrates^6–9^.

During the melt season, the colour of snow surface changes into red, green, golden-brown or orange due to snow algae blooming. Most common genera for each variety of the coloured snow are *Sanguina* for red snow, *Chloromonas* for green and orange snow, and *Ochromonas* for golden-brown snow. Species composition of snow algae varies across each coloured snow^5–7^, which drives different composition of other heterotrophic organisms^4^. Although a large number of taxonomic studies have been conducted only on snow algae, being primary producers sustaining ecosystems and affecting the reduction of snow albedo^9–13^, less attention has been paid to top consumers like tardigrades^4^. These organisms seem to be a forgotten faunal element in studies on snow ecosystems. Therefore, the description of faunal diversity and understanding whether snow ecosystems support any typical snow metazoans, which might be endangered due to global warming, is a crucial task.

Tardigrada (water bears) are a cosmopolitan phylum of microinvertebrates (mostly < 1 mm) that can live in almost all types of environments such as marine and terrestrial, from polar regions to the tropics^14–16^. Until now, ca. 1400 species of tardigrades have been reported^17^. However, an increasing number of new taxa descriptions is a robust indicator of many water bears still awaiting discovery across the globe^17^ (https://www.tardigrada.net). Owing to the ability of cryptobiosis that is a latent state under which tardigrade metabolism is undetectable, many limnoterrestrial species can withstand unfavourable conditions e.g., freezing^18,19^, high pressure^20^ and radiation^21,22^. However, active tardigrade species are found in extreme habitats such as ice or snow^4,16^. Tardigrades play multitrophic roles in ecosystems i.e. some of them may effectively control the population of other metazoans in soil ecosystems, tardigrades on snow feed on algae^4^. The question how many species inhabit snow ecosystems and how they differ from other tardigrades remains open.

New taxa of tardigrades in cold environments, like glaciers and ice sheets, have been described in recent years. For example, *Cryoconicus* with dark-brown pigment and claws of *Ramazzotius* type were reported from cryoconite granules (dark, oval, biogenic structures on ice; mixture of organic and mineral particles^23,24^) in glaciers of central Asia^25^, or *Cryobiotus* with dark pigment, big eyes and modified claws of *Hypsibius* type from cryoconite holes (water-filled reservoirs on glacial ice^26,27^) in mountainous glaciers of Europe and Asia^28–31^. These glacier genera have dark-coloured pigment, which is thought to protect from a high dose of UV radiation^25,32^. Cryoconite holes in the Arctic and Antarctica are also inhabited by transparent tardigrades, some of them representing new species, most probably glacier obligates^33^. Extensive field sampling revealed that some glacier tardigrades are unique and adapted to live only on glaciers^34^. Taking into account all findings from glacial ecosystems and recent findings of tardigrades on snow^4,35^, we decided to identify and provide the description of snow tardigrades to give a baseline for answering a question whether as on glaciers, tardigrades on snow are represented by unique, snow-requiring species.

In this paper, we report two taxa of tardigrades belonging to the Hypsibiidae family from snow ecosystems in Japan, one of which is formally described as a new species. We have analyzed green snow samples collected over two seasons (2019 and 2020) from seasonal snow patches at 750 m a.s.l. in Mt. Gassan in the north of Japan where active tardigrades have already been observed^4^. The first tardigrade found was described by classical methods combining light and scanning electron microscopy imaging, the second was diagnosed by combing light imaging with sequencing of nuclear and mitochondrial DNA fragments, two conservative (18S rRNA, 28S rRNA) and one more variable (COI).

## Results and discussion

**Figure 1:**
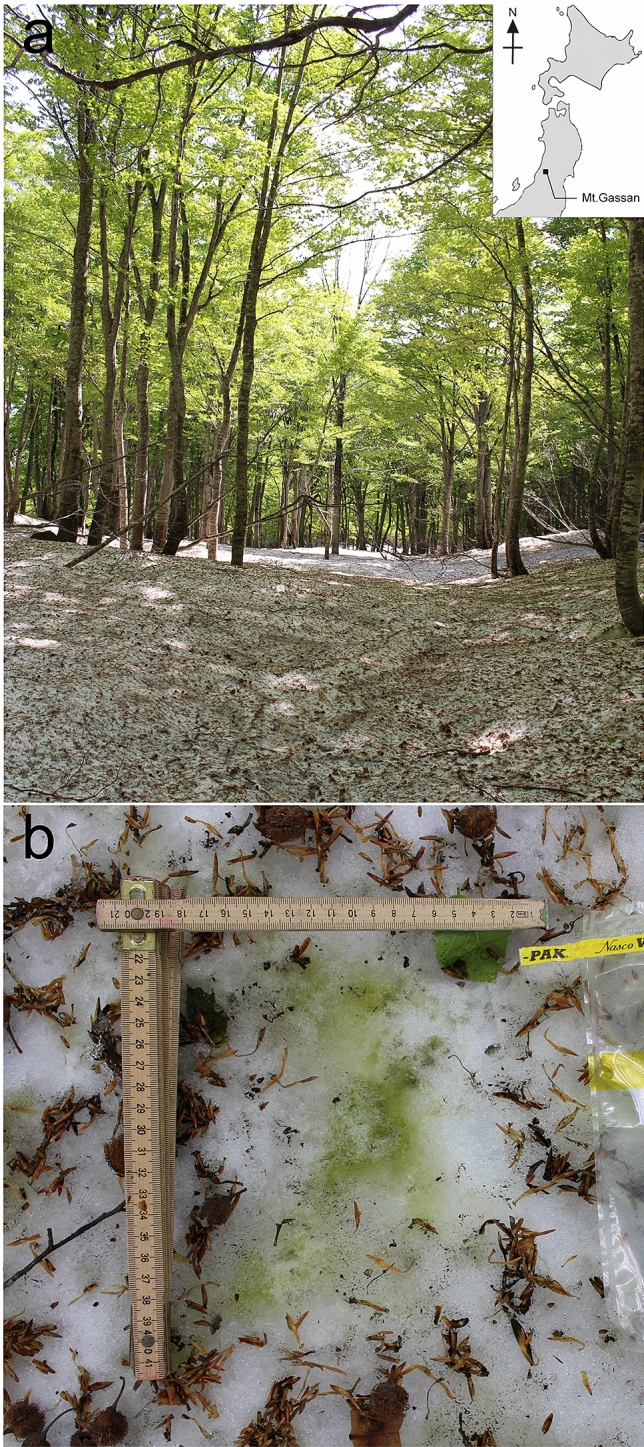
(**a**) Sampling area in May, 2019. Leaf of beech trees have already been opened. (**b**) Green snow patches collected in May, 2019.

**Figure 2:**
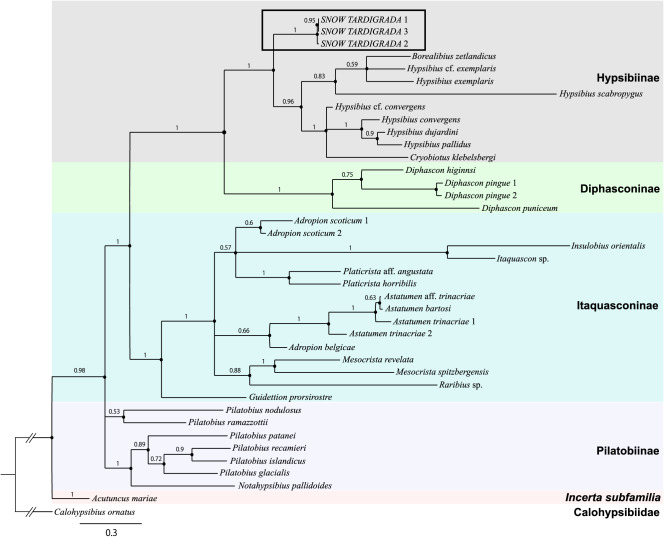
The phylogenetic position of *Hypsibius* sp. from Mt. Gassan (clade in the frame) in the Bayesian tree constructed from concatenated nucleotide sequences of the three molecular markers, one mitochondrial (COI) and two nuclear (18S rRNA, 28S rRNA). Support values are presented at the nodes.

We identified two taxa of tardigrades found in the blooming of green algae (*Chloromonas* spp.) on snow surface in Japan (Fig. 1a,b). Their intestine were green which suggest that they feed on *Chloromonas* spp. Both taxa belong to the one of the most species-rich tardigrade family of Hybsibiidae. According to morphology, both species belong to the Hypsiibinae subfamily. By morphology alone, we described here *Hypsibius* *nivalis* sp. nov., and by morphology and DNA we diagnosed the second taxon *Hypsibius* sp., which according to its morphology (claws of *Hypsibius* type, two macroplacoids, the lack of cuticular bars, hook-shaped AISM) belongs to the *convergens-pallidus* group. However, phylogenetic analysis (Bayesian) based on concatenated mitochondrial (COI) and nuclear (18S rRNA, 28S rRNA) molecular markers placed *Hypsibius* sp. from Mt. Gassan as a sister clade to Hypsibiinae subfamily (Fig. 2). This taxon was found by DNA during two sampling seasons in 2019 and 2020, which suggests its link with green algae blooming on the snow surface.

### Taxonomic account

Phylum: Tardigrada Doyère, 1840^36^

Class: Eutardigrada Richters, 1926^37^

Order: Parachela Schuster et al., 1980^38^

Superfamily: Hypsibioidea Pilato, 1969^39^

Family: Hypsibiidae Pilato, 1969^39^

Subfamily: Hypsibiinae Pilato, 1969^39^

Genus: *Hypsibius* Ehrenberg, 1848^40^

**Figure 3:**
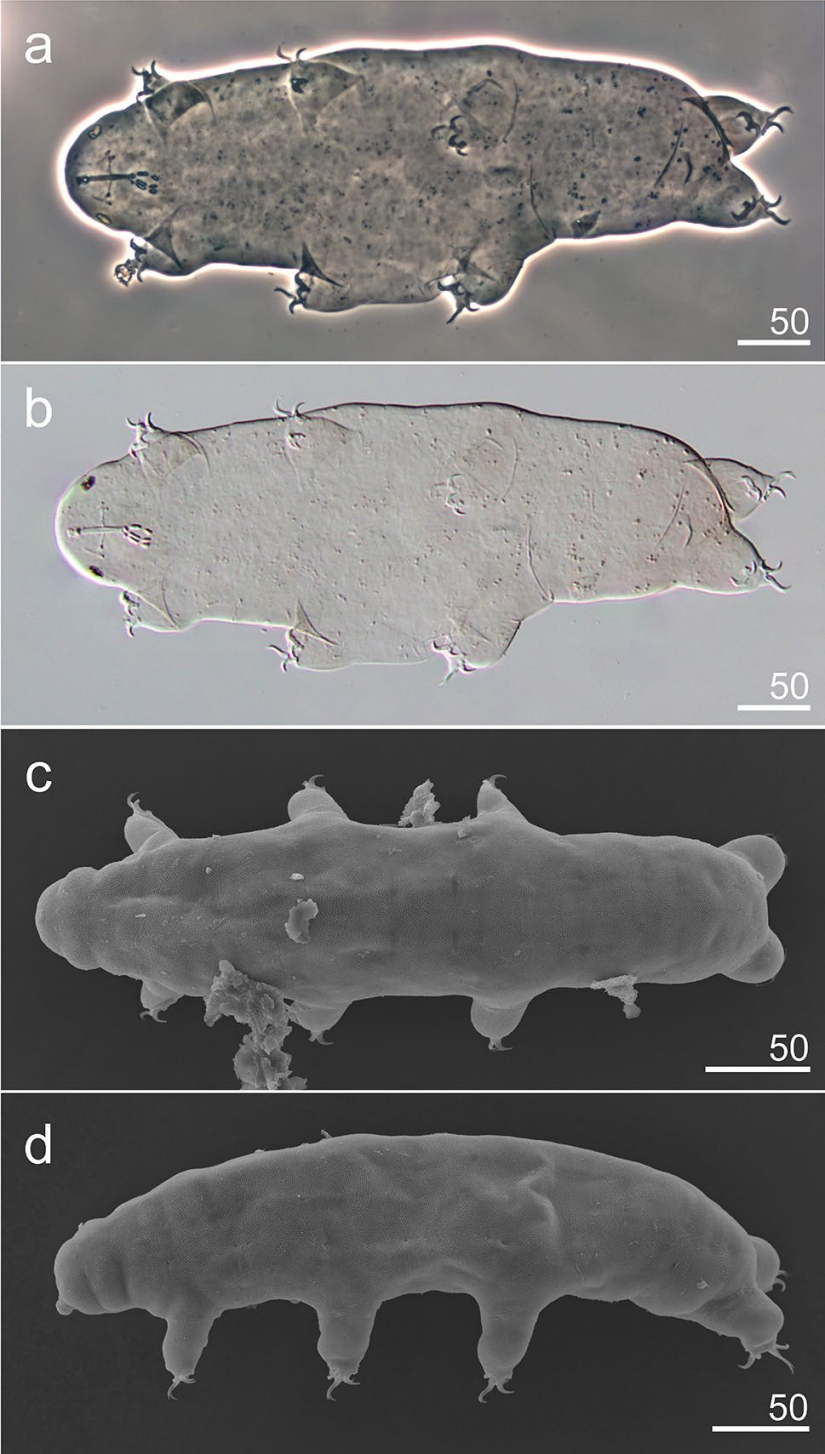
*Hypsibius* *nivalis* sp. nov., habitus: (**a**) ventrolateral view, holotype (PCM), (**b**) ventrolateral view, holotype (DIC), (**c**) dorsal view, paratype (SEM), (**d**) lateral view, paratype (SEM).

**Figure 4:**
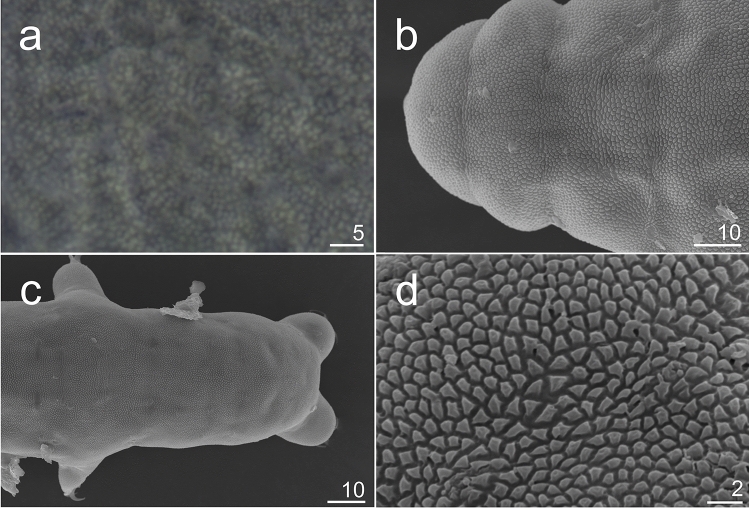
*Hypsibius* *nivalis* sp. nov., cuticular sculpture: (**a**) dorsal view, paratype (PCM), (**b**) dorsal view, cephalic part, paratype (SEM), (**c**) dorsal view, caudal part, paratype (SEM), (**d**) dorsal view, granular and separated polygons, paratype (SEM).

**Figure 5:**
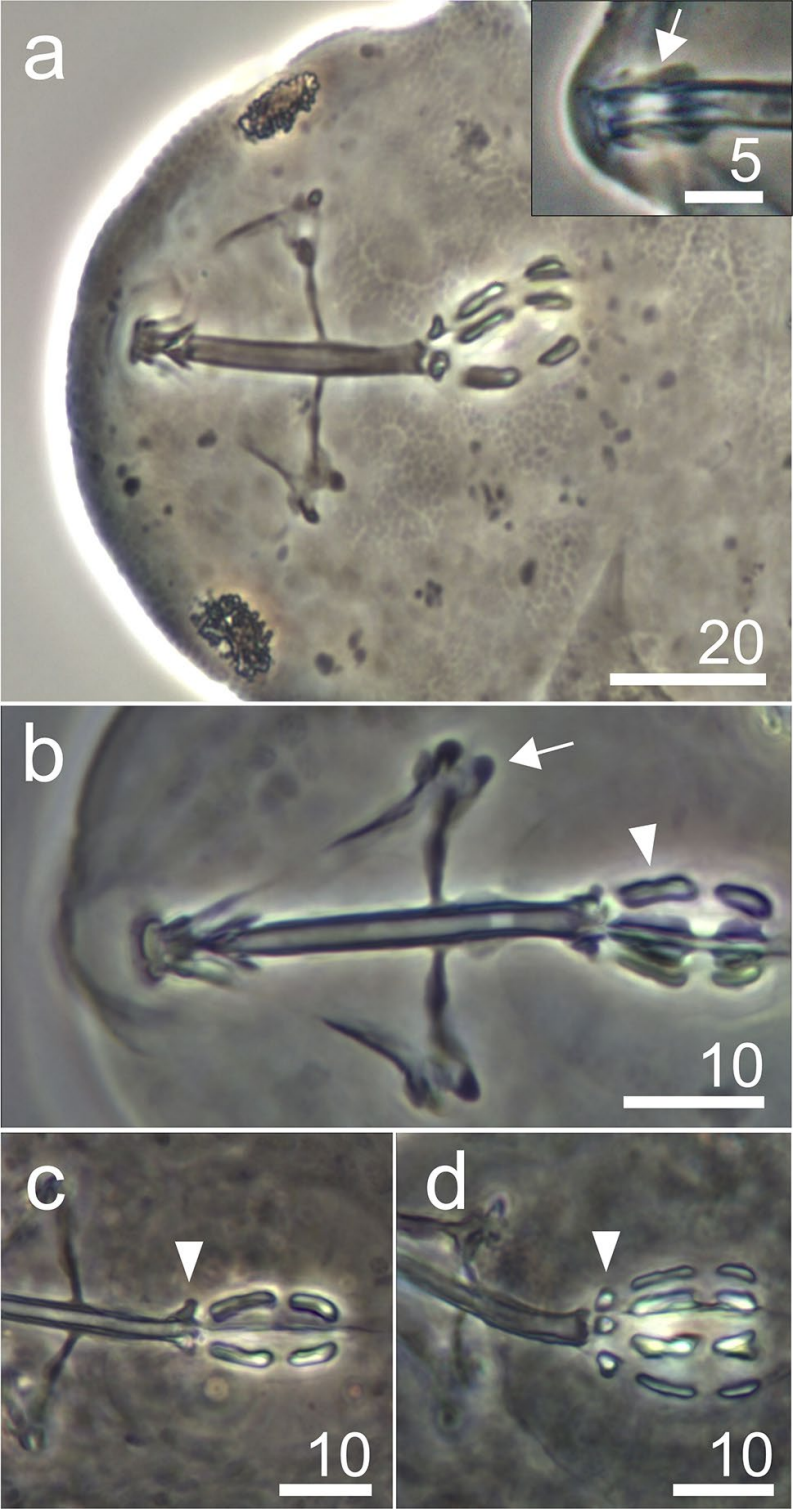
*Hypsibius* *nivalis* sp. nov., bucco-pharyngeal structures (PCM): (**a**) cephalic part, buccal apparatus and eyes, holotype, insert are AISM, (**b**) buccal apparatus, arrow indicates typical *Hypsibius* type furcae, arrowhead indicates incision in the first macroplacoid, paratype, (**c,d**) apophyses (arrowhead) and macroplacoids.

**Figure 6:**
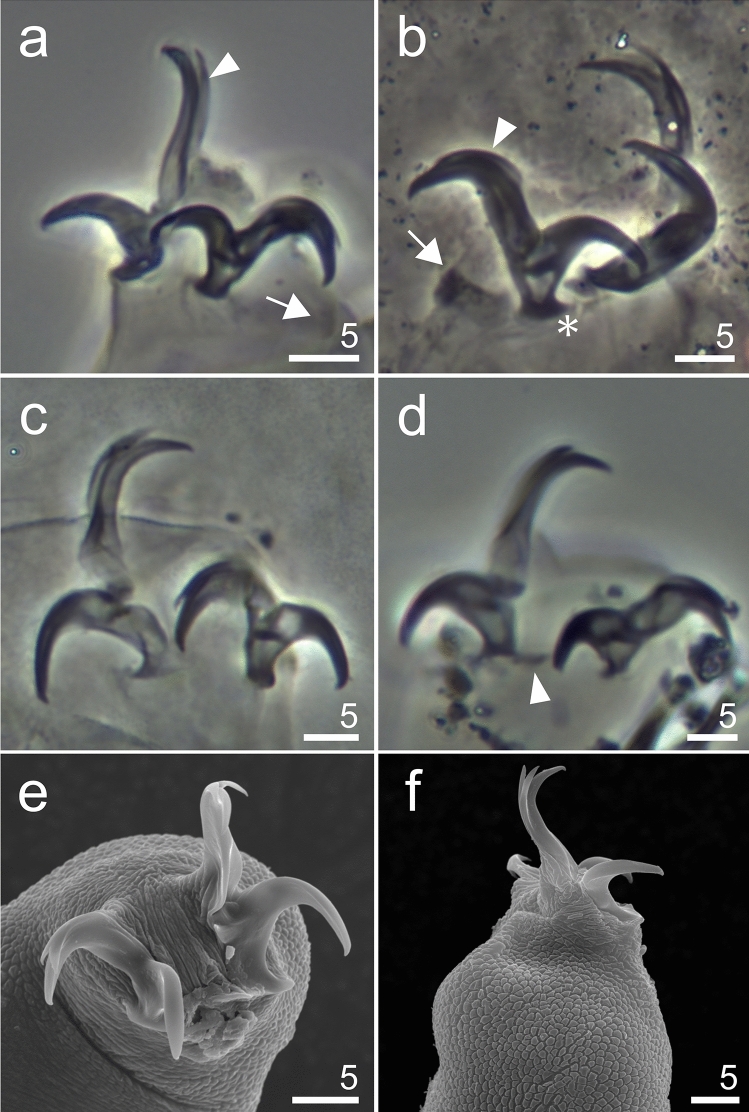
*Hypsibius* *nivalis* sp. nov., claws: (**a**) claws I, arrowheads indicates accessory points, arrow indicates cuticular bar, paratype (PCM), (**b**) claws II, arrowheads indicates accessory points, arrow indicates cuticular bar, asterix indicates pseudolunules, paratype (PCM), (**c**) claw III, paratype (PCM), (**d**) claws IV, arrowhead indicates small cuticular bar, holotype (PCM), (**e**) claws IV, details of claws and claw bases, paratype (SEM), (**f**) leg III, external claw, paratype (SEM).

***Hypsibius*** ***nivalis*** **sp. nov**. (Figs. 3, 4, 5 and 6, Table 1, Supplementary material 1).

**Table 1:** Measurements (in µm) of selected morphological structures of *Hypsibius* *nivalis* sp. nov., individuals mounted in Hoyer’s medium (N—number of the measured structures; range—the smallest and the largest measurements of the structure, SD—standard deviation). The *pt* values are in italic.

Character	N	Range						Mean		SD		Holotype	
		µm			*pt*			µm	*pt*	µm	*pt*	µm	*pt*
Body length	21	204	–	543				393		93		460	
**Buccopharyngeal tube**													
Buccal tube length	24	21.2	–	36.5		–		28.4	*–*	3.8	*–*	36.5	*–*
Stylet support insertion point	23	13.6	–	23.2	*58.4*	*–*	*67.8*	17.8	*63.0*	2.6	*2.5*	22.9	*62.7*
Buccal tube external width	28	2.6	–	4.6	*8.8*	*–*	*15.4*	3.1	*11.2*	0.4	*1.7*	4.0	*11.0*
Buccal tube internal width	28	1.0	–	2.8	*4.0*	*–*	*11.1*	1.6	*6.1*	0.5	*1.6*	2.3	*6.3*
**Placoid lengths**													
Macroplacoid 1	30	3.9	–	6.8	*14.4*	*–*	*23.2*	5.2	*18.5*	0.8	*2.0*	6.8	*18.6*
Macroplacoid 2	30	2.5	–	5.8	*9.6*	*–*	*16.8*	3.8	*13.5*	0.8	*1.8*	5.8	*15.9*
Macroplacoid row	30	8.4	–	14.7	*34.4*	*–*	*43.0*	10.7	*38.3*	1.4	*2.7*	14.7	*40.3*
**Claw 1 heights**													
External base	17	4.4	–	7.1	*15.9*	*–*	*23.0*	5.7	*19.8*	0.8	*2.2*	7.0	*19.2*
External primary branch	10	8.1	–	14.0	*33.8*	*–*	*42.2*	11.1	*38.1*	1.7	*3.2*	14.0	*38.4*
External secondary branch	17	5.2	–	9.0	*22.6*	*–*	*31.8*	7.3	*25.7*	1.0	*2.5*	9.0	*24.7*
Internal base	12	4.4	–	7.3	*14.9*	*–*	*20.0*	5.2	*18.1*	0.9	*1.6*	7.3	*20.0*
Internal primary branch	12	6.5	–	10.0	*22.0*	*–*	*28.5*	7.6	*25.9*	1.2	*1.9*	10.0	*27.4*
Internal secondary branch	13	4.8	–	8.7	*17.6*	*–*	*24.2*	6.2	*21.2*	1.2	*2.1*	8.7	*23.8*
**Claw 2 heights**													
External base	13	4.6	–	7.6	*17.2*	*–*	*23.7*	6.1	*20.6*	0.9	*1.9*	7.6	*20.8*
External primary branch	12	9.3	–	15.2	*37.7*	*–*	*43.4*	12.0	*39.9*	1.6	*1.9*	15.2	*41.6*
External secondary branch	12	5.1	–	9.2	*20.1*	*–*	*27.8*	7.3	*24.7*	1.1	*2.4*	9.2	*25.2*
Internal base	13	4.0	–	7.4	*16.4*	*–*	*22.0*	5.4	*18.7*	0.9	*1.6*	7.1	*19.5*
Internal primary branch	11	6.5	–	11.1	*25.5*	*–*	*31.2*	8.2	*28.6*	1.5	*2.1*	11.1	*30.4*
Internal secondary branch	12	4.8	–	9.1	*18.4*	*–*	*25.5*	6.5	*22.1*	1.3	*2.4*	9.1	*24.9*
**Claw 3 heights**													
External base	14	5.5	–	8.8	*18.9*	*–*	*26.8*	6.7	*23.1*	1.1	*2.8*	8.5	*23.3*
External primary branch	13	10.1	–	15.0	*36.4*	*–*	*44.7*	12.0	*40.9*	1.7	*2.8*	15.0	*41.1*
External secondary branch	14	6.2	–	11.4	*24.4*	*–*	*32.9*	8.1	*28.0*	1.4	*2.7*	11.4	*31.2*
Internal base	13	4.0	–	7.9	*14.9*	*–*	*23.4*	5.3	*18.3*	1.2	*2.8*	7.9	*21.6*
Internal primary branch	10	5.4	–	9.7	*20.1*	*–*	*29.4*	7.0	*24.6*	1.5	*3.6*	9.7	*26.6*
Internal secondary branch	12	4.3	–	8.8	*16.0*	*–*	*25.6*	6.2	*21.5*	1.4	*3.2*	8.8	*24.1*
**Claw 4 heights**													
Anterior base	12	3.8	–	8.0	*17.4*	*–*	*21.9*	5.7	*19.9*	1.2	*1.7*	8.0	*21.9*
Anterior primary branch	11	6.2	–	12.7	*28.4*	*–*	*34.8*	9.3	*32.4*	2.0	*1.9*	12.7	*34.8*
Anterior secondary branch	12	4.7	–	10.9	*21.2*	*–*	*29.9*	7.1	*24.9*	1.8	*3.0*	10.9	*29.9*
Posterior base	13	5.1	–	8.7	*20.5*	*–*	*26.8*	6.4	*22.9*	1.1	*2.0*	8.5	*23.3*
Posterior primary branch	13	10.5	–	18.8	*48.2*	*–*	*55.8*	14.4	*52.5*	2.4	*3.2*	17.7	*48.5*
Posterior secondary branch	13	6.3	–	12.4	*25.7*	*–*	*36.8*	8.5	*31.0*	2.0	*3.9*	12.2	*33.4*

**Type locality.** Japan, Mt. Gassan (38º 30′ N, 140º 00′ E: altitude 770 m a.s.l.).

**Type material.** Holotype, slide code: “April, 19, Japan snow no. 1/10” is deposited at the Graduate School of Science and Engineering, Chiba University, Chiba, Japan; 31 paratypes, slide codes: “April, Japan snow no. 1/4, 1/6–1/9, 1/15, 2/1–2/2, R/2, R/4–R6”; SEM stubs codes: “2005GA no. R-1, R” are deposited at the Graduate School of Science and Engineering, Chiba University, Chiba, Japan; and four paratypes, slide codes: “Japan snow 2/1, 2/3, 3/2” are deposited at Department of Animal Taxonomy and Ecology, Adam Mickiewicz University in Poznań.

**Etymology.** Name *nivalis* refers to the environment where the species was found –*nival* in a latin means related to snow.

**Description.** Body transparent/white, eyes present in all specimens mounted in Hoyer’s medium (Fig. 3a,b). Eyes composed of small granules (Figs. 3a,b,5a). Dorsal cuticle sculptured, covered by polygonal granules, each polygon is separated, polygons form reticular network (Fig. 4a–d). Polygons small in size, do not exceed 2 µm (Fig. 4d). Reticulum covers the legs dorsally. Ventral cuticle smooth. Buccal tube short and rigid (Fig. 5a–d). Teeth in the oral cavity armature absent or not visible under PCM. AISM blunt hook-shaped (Fig. 5a), similar to *Mixibius*^41^. Stylet supports located in posterior position of the buccal tube. Typical *Hypsibius* type stylet furcae (Fig. 5a,b). Pharynx with apophyses and with two rod-shaped macroplacoids. Apophyses are big, triangular or square in shape, clearly separated from macroplacoids. Macroplacoid length sequence 2 < 1. The first macroplacoid with constriction, clearly separated from the second one. Microplacoid and septulum absent (Fig. 5a–d). Claws of the *Hypsibius*-type, internal and anterior claws smaller than external and posterior claws respectively (Fig. 6a–f). Claws with widened bases and with obvious accessory points on the primary branches. Near the border between accessory points and primary claw branch, a thick line is visible along entire branch length (Fig. 6a–d). Smooth, indistinct pseudolunules under claws more visible on external claws (Fig. 6b). Claw bases smooth. Wide, cuticular bars at the internal claws I–III present, a small bar is present at the posterior claw IV (Fig. 6a–d). Eggs unknown.

**Differential diagnosis**. By having two macroplacoids, no microplacoid and septulum, and presence of cuticular sculpture, *Hypsibius* *nivalis* sp. nov. is the most similar to *Hypsibius* *biscuitiformis* Bartoš, 1960^42^, *Hypsibius* *calcaratus* Bartoš, 1935^43^, *Hypsibius* *camelopardalis* Ramazzotti & Maucci, 1983^44^, *Hypsibius* *giusepperamazzotti* Sudzuki, 1975^45^, *Hypsibius* *macrocalcaratus* Beasley, 1988^46^, *Hypsibius* *maculatus* Iharos, 1969^47^, *Hypsibius* *morikawai* Ito, 1995^48^, *Hypsibius* *ragonesei* Binda & Pilato, 1985^49^, *Hypsibius* *roanensis* Nelson & McGlothlin, 1993^50^, *Hypsibius* *runae* Bartoš, 1941^51^ and *Hypsibius* *stiliferus* Abe, 2004^52^ but differs from:

- *H.* *biscuitiformis* described from mosses in Hungary by: type of cuticular sculpture (polygonal granules, each polygon is separated, polygons form reticular network in *H.* *nivalis* sp. nov. *vs.* fine and regular granulation in *H.* *biscuitiformis*), presence of cuticular bars, and different shape of second macroplacoid (rod shaped in *H.* *nivalis* sp. nov. *vs.* granular macroplacoid in *H.* *biscuitiformis*).
- *H.* *calcaratus* described from Slovakia by: presence of cuticular bars, shape of claws (*convergens-pallidus* type in *H.* *nivalis* sp. nov. *vs.* *Ramazzottius* type in *H.* *calcaratus* (based on original drawings)), and wider buccal tube diameter (2.6–4.6 µm (external wide) in *H.* *nivalis* sp. nov. *vs.* 1–2 µm in *H.* *calcaratus*).
- *H.* *camelopardalis* described from Iberian Peninsula by: type of sculpture (polygonal granules, each polygon is separated, polygons form reticular network in *H.* *nivalis* sp. nov. *vs.* plates of various sizes in *H.* *cameopardialis*), presence of similar in size granular polygons on entire dorsal side of the body, presence of cuticular bars under the claws.
- *H.* *giusepperamazzotti* described from Tama River in Japan by: different macroplacoid length sequence (2 < 1 in *H.* *nivalis* sp. nov. *vs.* 1 < 2 in *H.* *giusepperamazzotti*), presence of cuticular bars under the claws.
- *H.* *macrocalcaratus* described from Mexico by: shape of macroplacoids (rod-shaped in *H.* *nivalis* sp. nov. *vs.* granular in *H.* *macrocalcaratus*), smaller cuticular granules (ca. 1–1.5 µm in *H.* *nivalis* sp. nov. *vs.* ca. 2 µm in *H.* *macrocalcaratus*), shape of claws (*convergens-pallidus* type in *H.* *nivalis* sp. nov. *vs.* *Ramazzottius* type in *H.* *macrocalcaratus*, based on original drawings and description in Beasley^46^).
- *H.* *maculatus* described from Cameroon by: type of cuticular sculpture (polygonal granules, each polygon is separated, polygons form reticular network in *H.* *nivalis* sp. nov. *vs.* hemispherical tubercles, arranged in transverse rows with many dark granules in *H.* *maculatus*), presence of cuticular bars, and relatively bigger body size (204–543 µm in *H.* *nivalis* sp. nov. vs*.* 200–225 µm in *H.* *maculatus*).
- *H.* *morikawai* described from mosses in Japan by: type of cuticular sculpture (polygonal granules, each polygon is separated, polygons form reticular network, well visible in PCM in *H.* *nivalis* sp. nov. vs. very faint rugulae in *H.* *morikawai*, according to the original description of Ito^48^).
  Note: Due to lack of detailed description and drawings of cuticular sculpture of *H.* *morikawai*, we analyzed holotype (courtesy provided by professor Masamichi Ito), we did not find similar cuticular pattern. We found only very faint shapes resembling cuticular sculpture (Supplementary material 2).
- *H.* *ragonesei* described from Italy by: type of cuticular sculpture (polygonal granules, each polygon is separated, polygons form reticular network in *H.* *nivalis* sp. nov. *vs.* wrinkled cuticle, distributed in bands on the dorsal side of the body), and presence of cuticular bars.
- *H.* *roanensis* described from lichens in Tenneesee (Roan Mountain) by: shape of macroplacoids (rod-shaped in *H.* *nivalis* sp. nov. *vs.* granular in *H.* *roanensis*), and presence of cuticular bars.
- *H.* *runae* described from Carpathians by: type of cuticular sculpture (polygonal granules, each polygon is separated, polygons form reticular network in *H.* *nivalis* sp. nov. *vs.* dorsal cuticle covered by papillae in *H.* *runae*) and presence of cuticular bars.
- *H.* *stiliferus* described from east Russia by: shape of macroplacoids (rod-shaped in *H.* *nivalis* sp. nov. *vs.* granular in *H.* *stiliferus*), and type of cuticular sculpture (polygonal granules, each polygon is separated, polygons form reticular network in *H.* *nivalis* sp. nov. *vs.* polygons of various size sparsely arranges in eight transverse rows in *H.* *stiliferus*), size of polygons (ca. 1–1.5 µm in *H.* *nivalis* sp. nov. *vs.* 0.8–4 µm in *H.* *stiliferus*), and presence of cuticular bars.

**Remarks.** Regrettably, the amplification of DNA fragments of *Hypsibius* *nivalis* sp. nov. failed (we did not have a fresh material for new analysis).

**Figure 7:**
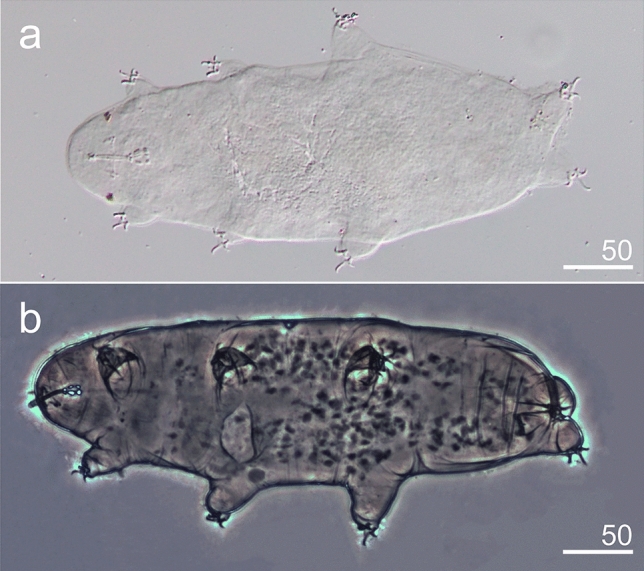
*Hypsibius* sp. from Mt. Gassan, habitus: (**a**) ventral view (DIC), (**b**) ventrolateral view (PCM).

**Figure 8:**
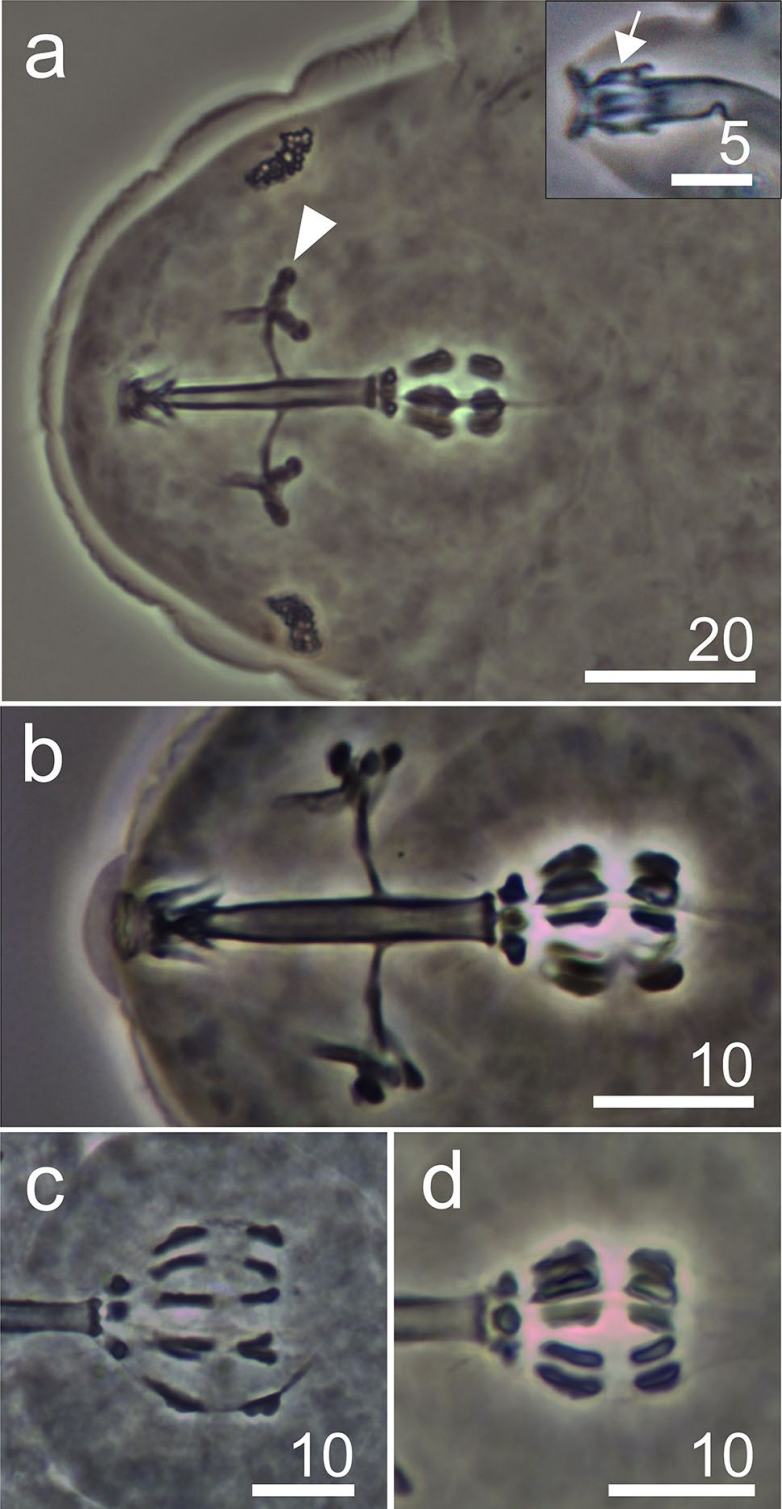
*Hypsibius* sp. from Mt. Gassan, bucco-pharyngeal structures (PCM): (**a**) cephalic part, buccal apparatus and eyes, arrowhead indicates typical *Hypsibius* type furcae, insert—AISM, (**b**) buccal apparatus, (**c,d**) macroplacoids and apophyses.

**Figure 9:**
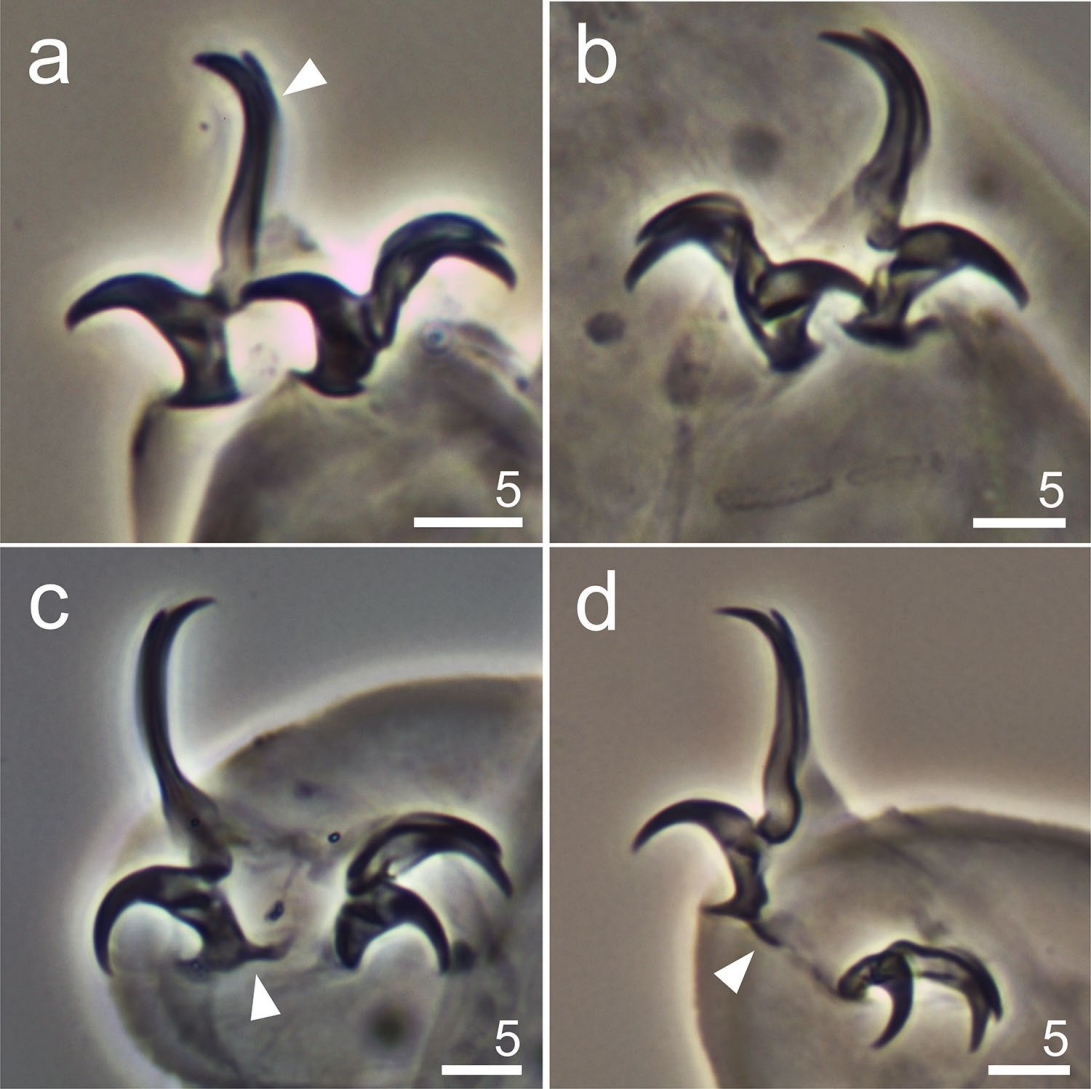
*Hypsibius* sp. from Mt. Gassan, claws (PCM): (**a**) claws I, arrowheads indicates accessory points, (**b**) claws II, (**c**) claws IV, arrowhead indicates widened posterior claw base, (**d**) claw IV, arrowhead indicates widened posterior claw base that form very faint connection between anterior and posterior claws.

***Hypsibius* sp.** from Mt. Gassan (Figs. 2, 7–9 , Supplementary material 3).

**Diagnosis.** The body transparent/white, with eyes present in the examined specimens. The cuticle smooth in the PCM (Fig. 7a,b). The buccal apparatus of the *Hypsibius* type (Fig. 8a,b). Oral cavity armature either absent or not visible in the PCM (Fig. 8b). The pharyngeal bulb with apophyses, with two rod-shaped macroplacoids (Fig. 8a–d). Stylet supports located in the posterior position. AISM hook-shaped, as presented for *Hypsibius* in Pilato^41^ (Fig. 8a). *Hypsibius* type furcae present. The macroplacoid length sequence 2 < 1, microplacoid and septulum absent (Fig. 8a–d). The apophyses clearly separated from the 1st macroplacoids. All macroplacoids clearly separated (Fig. 8c,d). All main branches with accessory points (Fig. 9a–d). Cuticular bars under and between the claws absent. However the thickening under the claw base IV clearly visible (Fig. 9c,d). Claw basess smooth. Proper lunulae absent, poorly visible pseudolunules present. Eggs unknown.

**Molecular delimitation.** The ASAP analysis of 13 COI sequences (11 of tardigrades from snow together with *H.* *dujardini* and *H.* *exemplaris*) identified 3 putative species at asap-score = 1 (one species of *Hypsibius* sp. from Mt. Gassan and other two *Hypsibius* species: *H.* *exemplaris* and *H.* *dujardini*, Supplementary material 4).

Intraspecific uncorrected pairwise distances for COI marker within 11 specimens of *Hypsibius* sp. from Mt. Gassan varied between 0 and 2.48% (Supplementary material 5). The p-distances calculated in Mega follow results of ASAP indicating one species of *Hypsibius* sp. from Mt. Gassan. The sequences of COI, 18S rRNA and 28S rRNA are deposited in GenBank under accession numbers: ON899873–ON899875, ON898549 and ON927924–ON927925, respectively.

**Remarks on the species.** This species belongs to a large group of hypsibiids with smooth cuticle, two macroplacoids and the lack of cuticular bars under the claws I-III^15,33,53^. Although phylogenetic analysis placed *Hypsibius* sp. from Mt. Gassan as a sister lineage to species of Hypsibiinae (Fig. 2), the formal erection of the species as a new taxa without integrative redescription of the most similar by morphology *Hypsibius* *convergens* and *Hypsibius* *pallidus*, made an exact morphological differential diagnosis dubious. As it has already been shown by using molecular approaches, the genus *Hypsibius* is polyphyletic, representing similar morphology but distant genetics among its taxon^53–55^. According to DNA, it could be erected as a separate genus, however morphological obstacles do not allow for a proper description in contrast to *Cryobiotus* or *Borealibius* which are nested among with other *Hypsibius* species but differ from them by morphology^29,56^. Therefore, we decided to only present data on the morphology, morphometry and DNA of a potentially new species from snow.

### Tardigrades on snow

Tardigrades have previously been found in snow called “Akashibo” in blooms of algae *Hemitoma* sp.^57^, and in red snow consisting of algae *Chloromonas* spp. and *Sanguina* spp. in North America^5^. In spite of that, they have not been identified. Here, we present the taxonomic description of tardigrades from snow for the first time. Our study shows that snow ecosystems are overlooked for studies on the diversity of microinvertebrates. Although tardigrades are most probably wind-blown, delivered to the snow surface in forests from tree canopies or tree trunks^58,59^, they establish a stable population in snow algae blooming, have persisted for multiple seasons, representing different instars and laying eggs^4^. Whether specific species of tardigrades need snow (a low-temperature habitat) for their growth and reproduction or they can be only found in a habitat providing them with food (green algae), and without a high number of competitors (compared to mosses) is an open question and requires future findings. Nevertheless, an increasing number of evidence indicates that tardigrades reproduce and feed on snow algae^4,5^. However, the fate of tardigrades from snow during summer time is unknown and both scenarios, tardigrades are active and reproduce on the snow as well as in mosses after snow melt cannot be ruled out. If these animals need snow ecosystems as a part of their life cycle as was suggested^4^, and like their glacial counterparts^34^, the global disappearance of snow ecosystems^2,60^ may trigger negative changes for snow algae blooming associated metazoans.

## Material and methods

### Sample processing

Snow sampling was conducted at Yumiharidaira park (38°30′N, 140°00′E: altitude 770 m above sea level (a.s.l.)) on Mt. Gassan, Yamagata prefecture in Japan (Fig. 1a), details on sampling sites are provided in Ono et al.^4^. Green snow samples were collected in April and May, 2019 and May, 2020 from seasonal snow in forest area surrounded by beech trees (Fig. 1b). The samples, dimension with 10 × 10 × 2 cm (length × width × depth), were collected using sterile stainless-steel scoop. After sampling, all the samples were kept frozen in Whirl–Pak^®^ bags (Nasco, Fort Atkinson, WI, USA). Tardigrades were isolated from the samples in September 2019 and December 2020, then fixed with 70% ethanol for preservation. Some specimens were mounted on permanent slides for imaging and morphometry in phase contrast light microscopy (PCM), differential interference contrast microscopy (DIC) or for scanning electron microscopy (SEM), remains were used for DNA sequencing.

### Microscopy and imaging

Specimens for phase contrast microscopy (PCM) and differential interference contrast microscopy (DIC) were mounted on microscope slides in a drop of Hoyer’s medium^61^ and examined under a microscopes Olympus BX51 (PCM) and BX53 (DIC). Pictures were taken with a DP21 digital camera (Olympus, Tokyo, Japan), cellSens Entry 1.12 software or Quick PHOTO CAMERA 3.0 software (Promicra, Prague, Czech Republic). Tardigrades for scanning electron microscopy (SEM) were processed following protocol of Sugiura et al.^62^ with some modifications. Specimens were washed with 0.1 M phosphate buffer, pH 7.0, and fixed with 4% glutaraldehyde, washed in phosphate buffer again and incubated in a 1% OsO_4_ solution for 1 h. Then specimens were washed with MiliQ water, and dehydrated in ethanol series 30%, 50%, 70%, 80%, 90%, 95%, and three times in 100% for 20 min. After maintained in 100% t-butyl alcohol for 3 h at refrigerator (5℃), specimens were lyophilized by using JFD-320 (JEOL, Tokyo, Japan), then coated with gold and examined using a scanning electron microscope JSM-6010PLUS/LA (JEOL, Tokyo, Japan).

### Morphometrics and nomenclature

Sample size for morphometrics was chosen following recommendations by Stec et al.^63^. All measurements are given in micrometers (μm) and were performed under PCM with Quick PHOTO CAMERA 3.0 software. Structures were measured only when their orientations were suitable. Body length was measured from the anterior to the posterior end of the body, excluding the hind legs. The *pt* ratio is the ratio of the length of a given structure to the length of the buccal tube, expressed as a percentage^64^. Macroplacoid length sequence was determined according to Kaczmarek et al.^65^. Claws were measured following Beasley et al.^66^. Tardigrade taxonomy and systematics is presented according to Bertolani et al.^67^ and Degma et al.^17^. Morphometric data were handled using the “Parachela” ver. 1.2 template available from the Tardigrade Register^68^. All microscope slides are deposited at the Graduate School of Science and Engineering, Chiba University, Chiba, Japan; and at Department of Animal Taxonomy and Ecology, Adam Mickiewicz University, Poznań, Poland.

### DNA extraction and amplification

Total genomic DNA was extracted individually from 11 specimens using the DNAeasy Blood and Tissue Kit (Qiagen GmbH, Hilden, Germany) according to the manufacture instruction. In order to get exoskeletons, after 48 h of digestion and then lysis, 300 ml of a mixture (i.e., ATL buffer—Qiagen, proteinase K, Lysis buffer—Qiagen and 96% ethyl alcohol) with a tardigrade in a 1.5 ml Eppendorf tube was centrifuged at 7000 min^−1^. Then, from each tube, 290 ml of the above mixture was carefully removed using a pipette remaining the tardigrade specimen on the bottom in 10 ml of the mixture. The exoskeleton was preserved and then mounted in Hoyer’s medium for morphological analysis.

A fragment of the cytochrome c oxidase subunit I (COI) gene of mtDNA was amplified with a bcdF01 forward primer (5'-CATTTTCHACTAAYCATAARGATATTGG-3') and bcdR04 reverse primer (5'-TATAAACYTCDGGATGNCCAAAAAA-3')^69,70^. A sequence of 18S rRNA gene of nDNA was amplified using the following primers 18s_Tar_1Ff (5'-AGGCGAAACCGCGAATGGCTC-3') and 18s_Tar_1Rr (5'-GCCGCAGGCTCCACTCCTGG-3')^71^. D1-D3 region of 28S rRNA gene nDNA was amplified with 28sEUTAR_F (5'-ACCCGCTGAACTTAAGCATAT-3')^53^ or 28sF0001 (5'-ACCCVCYNAATTTAAGCATAT-3')^72^ forward and 28sR0990 (5'-CCTTGGTCCGTGTTTCAAGAC-3')^72^ reverse primers.

Amplification of 18S rRNA and 28S rRNA nucleotide genes fragments was conducted in a total volume of 10 ml including 5 ml Type-it Microsatellite PCR Kit (Qiagen), 0.5 ml of each primer (10 ng ml^−1^), 0.5 ml Q-Solution (Qiagen) and 3.5 ml of the DNA template. For the COI gene, a total volume of 5 ml was prepared, including 3 ml Type-it Microsatellite PCR Kit (Qiagen), 0.5 ml of each primer (10 ng ml^−1^) and 1 ml of the DNA template. For amplification 18S rRNA and 28S rRNA gene fragments, a thermocycling profile with one cycle of 5 min at 95 °C followed by 38 steps of 30 s each at 95 °C, 60 s at 60 °C, 1 min at 72 °C, and with a final elongation of 5 min at 72 °C was used. While for COI gene fragment amplification, a hybridization was done at 50 °C for 1 min. After amplification, the PCR products were diluted double-fold with RNase-Free water, after that the diluted PCR product was analysed by electrophoresis on 1% agarose gel. Samples containing visible uniform bands with the expected length of the product were purified with Exonuclease I and Fast alkaline phosphatase (Fermentas). The samples were sequenced using the BigDye Terminator v3.1 kit and the ABI Prism 3130xl Genetic Analyzer (Applied Biosystems), following the manufacturer’s instructions.

### Phylogeny

The phylogenetic analyses were conducted using concatenated nuclear (18S rRNA, 28S rRNA) and mitochondrial (COI) sequences of 35 Hypsibiidae taxa, one representative of Incerta subfamilia (similar in morphology to *Hypsibius* sp. from Mt. Gassan genus *Acutuncus*) with *Calohypsibius* *ornatus*^73^ as the outgroup to hypsibiids. The phylogenetic pipline follows that of recently published robust phylogenies^74–77^. Sequences were downloaded from GenBank and the full list of accession numbers is given within Supplementary Material 6.

The sequences were aligned using the AUTO method (in the case of COI) and the Q-INS-I method (18S rRNA and 28S rRNA) in MAFFT version 7^78,79^. Then, the aligned sequences were trimmed to: 657 (18S rRNA), 335 (28S rRNA), 490 (COI) bp. All COI sequences were translated into protein sequences in MEGA7 version 7.0^80^ to check against pseudogenes. The sequences were then concatenated in SequenceMatrix^81^. Using PartitionFinder version 2.1.1^82^ under the Akaike Information Criterion (AIC), and with greedy algorithm^83^ implemented within the software we chose the best scheme of partitioning and substitution models for posterior phylogenetic analysis. As the COI is a protein coding gene, before partitioning, we divided our alignment of this marker into 3 data blocks constituting separated three codon positions. Best-fit partitioning schemes and models suggested by PartitionFinder are given within Supplementary Material 7.

Bayesian inference (BI) marginal posterior probabilities were calculated using MrBayes v3.2^84^. Random starting trees were used and the analysis was run for fifteen million generations, sampling the Markov chain every thousand generations. An average standard deviation of split frequencies of < 0.01 was used as a guide to ensure the two independent analyses had converged. The program Tracer v1.6^85^ was then used to ensure Markov chains had reached stationarity and to determine the correct ‘burn-in’ for the analysis, which was the first 10% of generations. The ESS values were greater than 200 and a consensus tree was obtained after summarising the resulting topologies and discarding the ‘burn-in’. All final consensus trees were viewed and visualised with FigTree v.1.4.3 available from (http://tree.bio.ed.ac.uk/software/figtree), then, the tree was modified in Adobe Illustrator, version 25.4.1.

### Species delimitation

The species were identified and compared with other taxa based on the previous descriptions^42,43,46,48,49,52^. If the information on the cuticular bars at the claws was not available either in original descriptions or drawings we assumed these structures were absent.

Using data sets for COI which includes sequences newly generated in this study, as well as COI sequences from *Hypsibius* *dujardini*^36^ and *Hypsibius* *exemplaris*^53^ we performed a genetic species delimitation analyses named the Assemble Species by Automatic Partitioning (ASAP)^86^. The analyses were run on the respective server (https://bioinfo.mnhn.fr/abi/public/asap/asapweb.html) with default settings. Additionally, we run analysis of p-distance in MEGA7 version 7.0^80^ for COI of *Hypsibius* sp. from Mt. Gassan.

## Supplementary Information

Supplementary Information 1.

Supplementary Information 2.

Supplementary Information 3.

Supplementary Information 4.

Supplementary Information 5.

Supplementary Information 6.

Supplementary Information 7.

## Data Availability

All morphometric data are available in the supplementary materials. The sequences of COI, 18S rRNA and 28S rRNA are deposited in GenBank under accession numbers: ON899873–ON899875, ON898549 and ON927924–ON927925, respectively. The slides are available at the Department of Animal Taxonomy and Ecology at Adam Mickiewicz University in Poznań and at the Graduate School of Science and Engineering, Chiba University, Chiba, Japan.
