# Supplementary material for: Description of a new species of Tardigrada Hypsibius nivalis sp. nov. and new phylogenetic line in Hypsibiidae from snow ecosystem in Japan
Source: Sci Rep. 2022 Sep 2;12:14995. doi: 10.1038/s41598-022-19183-8 (PMC9440035; doi:10.1038/s41598-022-19183-8)

Supplementary Figure for Ono *et al*. "*Description of a new species of Tardigrada Hypsibius nivalis sp. nov. and new phylogenetic line in Hypsibiidae from snow ecosystem in Japan*".

Supplementary material 1, Figure S1. *Hypsibius morikawai*, holotype: (a) habitus, (b) buccal tube with macroplacoids, (c) claws III, (d) claws IV, (e-g) cuticle with very faint pattern, that is most probably located under cuticle and doesn't represent typical cuticular sculpturing. Scale bars are in micrometres.

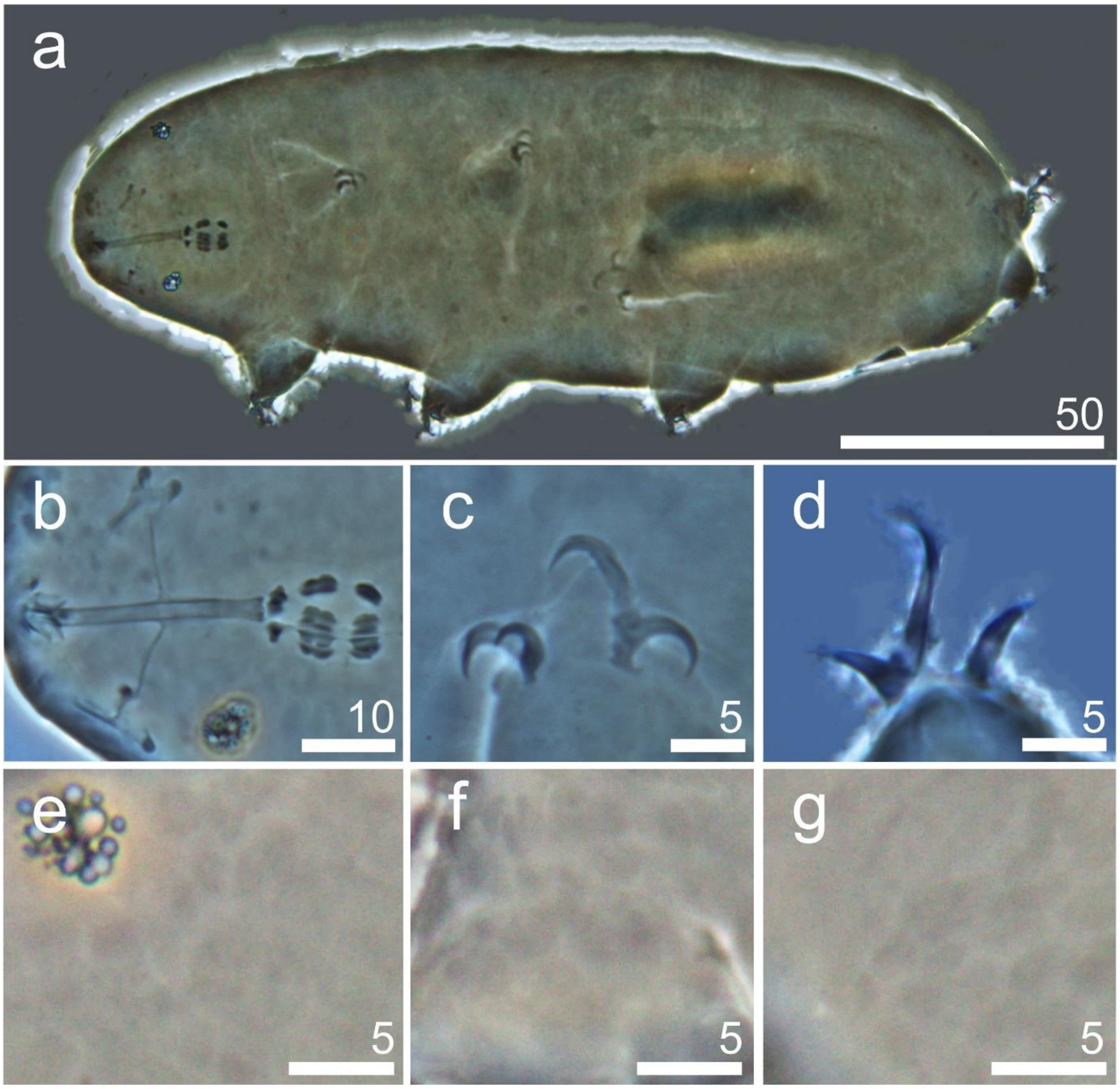

Supplement: Supplementary file 2 — Supplementary Information 2. [file 41598_2022_19183_MOESM2_ESM.pdf]
