# Supplementary material for: Description of a new species of Tardigrada Hypsibius nivalis sp. nov. and new phylogenetic line in Hypsibiidae from snow ecosystem in Japan
Source: Sci Rep. 2022 Sep 2;12:14995. doi: 10.1038/s41598-022-19183-8 (PMC9440035; doi:10.1038/s41598-022-19183-8)
Supplement: Supplementary file 3 — Supplementary Information 3. [file 41598_2022_19183_MOESM3_ESM.pdf]

Supplementary material 1, Table S1. Measurements (in µm) of selected morphological structures of *Hypsibius* sp. from Mt. Gassan, individuals mounted in Hoyer's medium (N - Number of the measured structures. Range - the smallest and the largest measurements of the structure, SD - standard deviation).

**CHARACTER**	**N**	**RANGE**						**MEAN**		**SD**	
		**µm**			***pt***			**µm**	***pt***	**µm**	***pt***
Body length	30	222	–	417	*935*	–	*1349*	330	*1205*	47	*90*
Buccopharyngeal tube											
Buccal tube length	30	22.2	–	34.7		–		27.4	–	3.4	–
Stylet support insertion point	30	13.8	–	20.6	*58.7*	–	*66.4*	17.1	*62.5*	2.0	*2.0*
Buccal tube external width	30	3.1	–	4.2	*9.8*	–	*16.0*	3.5	*12.8*	0.3	*1.7*
Buccal tube internal width	30	1.6	–	2.8	*5.5*	–	*10.0*	2.2	*8.0*	0.2	*1.2*
Placoid lengths											
Macroplacoid 1	29	3.2	–	6.6	*13.1*	–	*22.1*	4.9	*17.6*	0.9	*2.0*
Macroplacoid 2	29	2.4	–	4.9	*9.8*	–	*16.4*	3.3	*11.9*	0.6	*1.6*
Macroplacoid row	29	8.1	–	13.1	*34.7*	–	*43.6*	10.8	*39.1*	1.4	*2.5*
Claw 1 heights											
External base	15	3.5	–	5.9	*12.4*	–	*19.6*	4.7	*17.0*	0.7	*1.9*
External primary branch	15	7.4	–	10.8	*28.2*	–	*38.0*	9.2	*33.6*	1.0	*2.8*
External secondary branch	15	4.9	–	8.1	*17.4*	–	*23.6*	5.9	*21.4*	0.9	*1.8*
External base/primary branch (cct)	15	42.0	–	56.4		–		50.7	–	4.7	–
Internal base	15	3.1	–	5.5	*10.4*	–	*19.3*	4.2	*15.4*	0.9	*2.1*
Internal primary branch	13	4.8	–	8.3	*18.8*	–	*26.7*	6.5	*23.6*	1.1	*2.3*
Internal secondary branch	14	3.3	–	6.6	*11.1*	–	*23.5*	5.1	*18.5*	1.2	*3.3*
Internal base/primary branch (cct)	13	54.9	–	69.6		–		64.1	–	4.8	–
Claw 2 heights											
External base	23	4.0	–	7.0	*16.0*	–	*23.1*	5.3	*19.4*	0.9	*2.2*
External primary branch	22	7.7	–	12.3	*31.4*	–	*41.3*	10.4	*37.5*	1.3	*2.9*
External secondary branch	22	4.9	–	8.3	*17.8*	–	*27.1*	6.4	*23.0*	1.0	*2.4*
External base/primary branch (cct)	22	43.8	–	58.0		–		51.4	–	4.8	–
Internal base	16	3.5	–	5.8	*13.3*	–	*21.2*	4.6	*16.4*	0.8	*2.1*
Internal primary branch	10	5.7	–	7.9	*21.5*	–	*26.9*	6.8	*24.8*	0.8	*1.6*
Internal secondary branch	16	4.8	–	6.8	*16.1*	–	*21.6*	5.5	*19.7*	0.7	*1.6*
Internal base/primary branch (cct)	9	55.4	–	64.1		–		61.7	–	2.8	–
Claw 3 heights											
External base	15	4.0	–	6.7	*16.7*	–	*21.5*	5.4	*19.3*	0.8	*1.6*
External primary branch	15	8.0	–	12.7	*30.8*	–	*41.6*	10.3	*37.2*	1.4	*3.4*
External secondary branch	15	5.1	–	7.6	*20.2*	–	*25.7*	6.3	*22.8*	0.8	*1.6*
External base/primary branch (cct)	15	46.4	–	58.9		–		52.2	–	3.6	–
Internal base	13	3.4	–	5.7	*15.1*	–	*20.1*	4.9	*17.3*	0.7	*1.6*
Internal primary branch	12	5.4	–	8.5	*20.0*	–	*27.3*	6.9	*24.3*	1.1	*2.1*
Internal secondary branch	14	4.1	–	7.0	*16.8*	–	*22.7*	5.5	*19.5*	0.9	*1.8*
Internal base/primary branch (cct)	11	60.6	–	76.1		–		69.6	–	5.8	–
Claw 4 heights											
Anterior base	14	2.9	–	5.9	*12.6*	–	*18.8*	4.3	*15.6*	0.8	*1.8*
Anterior primary branch	14	5.4	–	9.8	*23.4*	–	*31.3*	7.5	*27.5*	1.2	*2.4*
Anterior secondary branch	14	3.3	–	7.1	*14.3*	–	*23.9*	5.3	*19.4*	1.0	*2.7*

Anterior base/primary branch (cct)	14	52.5	–	62.7		–		56.8	–	3.2	–
Posterior base	21	4.0	–	6.9	*16.7*	–	*23.7*	5.5	*19.7*	0.9	*1.9*
Posterior primary branch	21	9.8	–	16.6	*40.9*	–	*51.4*	13.0	*46.6*	2.2	*3.5*
Posterior secondary branch	21	5.3	–	9.3	*21.8*	–	*29.9*	7.3	*26.1*	1.1	*2.3*
Posterior base/primary branch (cct)	21	37.3	–	47.9		–		42.4	–	3.1	–
