# Supplementary material for: Description of a new species of Tardigrada Hypsibius nivalis sp. nov. and new phylogenetic line in Hypsibiidae from snow ecosystem in Japan
Source: Sci Rep. 2022 Sep 2;12:14995. doi: 10.1038/s41598-022-19183-8 (PMC9440035; doi:10.1038/s41598-022-19183-8)
Supplement: Supplementary file 6 — Supplementary Information 6. [file 41598_2022_19183_MOESM6_ESM.pdf]

Supplementary material 6. List of the GenBank accession numbers for hypsibiid sequences (18S r RNA, 28S rRNA, COI,) used for phylogenetic analyses of *Hypsibius* sp. from Mt. Gassan.

Species	18S	28S	COI	Reference
Acutuncus mariae	MW012739	MW012742	MW009690	Zawierucha et al. (2020)
Adropion scoticum voulcher 5	MT126752	MT126770	MT107466	Gąsiorek and Michalczyk (2020)
Adropion scoticum voucher 6	MT126753	MT126771		Gąsiorek and Michalczyk (2020)
Astatumen aff trinacriae	MT126755	MT126773	MT107468	Gąsiorek and Michalczyk (2020)
Astatumen bartosi	MT126754	MT126772	MT107467	Gąsiorek and Michalczyk (2020)
Astatumen trinacriae 1	HQ604922			Bertolani et al. (2014)
Astatumen trinacriae 2	FJ435733	FJ435775		Guil and Giribet (2012)
Borealibius zetlandicus	HQ604924			Bertolani et al. (2014)
Calohypsibius ornatus	MH279652	MT126784		Gąsiorek et al. (2019)
Adropion belgicae	HQ604925			Bertolani et al. (2014)
Diphascon higinnsi	HQ604932			Bertolani et al. (2014)
Pilatobius nodulosus	HQ604934			Bertolani et al. (2014)
Pilatobius patanei	HQ604935			Bertolani et al. (2014)
Diphascon pingue	HQ604937			Bertolani et al. (2014)
Diphason pingue	FJ435736	FJ435776		Guil and Giribet (2012)
Diphascon puniceum	EU266948			Sands *et al.,* (2008)

Pilatobius ramazzottii	HQ604939			Bertolani et al. (2014)
Guidettion prorsirostre	MT126750	MT126768	MT107464	Gąsiorek and Michalczyk (2020)
Hypsibius cf. *exemplaris*			MW010376	Zawierucha et al. (2020)
Hypsibius cf. *convergens*	KC582832			Dabert et al. (2013)
Hypsibius convergens	FJ435726			Guil and Giribet (2012)
Hypsibius dujardini	MG777532	MG777533	MG818723	Gąsiorek et al. (2018)
Hypsibius exemplaris	MG800327	MG800337	MG818724	Gąsiorek et al. (2018)
Cryobiotus klebelsbergi	KT901828	KT901829	KT901833	Dabert et al. (2015)
Notahypsibius pallidoides	MK973069	MK967963	MN919385	Tumanov (2020)
Hypsibius pallidus	HQ604945			Bertolani et al. (2014)
Hypsibius scabropygus	KC582831			Dabert et al. (2013)
Insulobius orientalis	MT126764	MT126783		Gąsiorek and Michalczyk (2020)
Itaquascon sp.	MT126759	MT126778	MT107473	Gąsiorek and Michalczyk (2020)
Mesocrista revalata	KU528627	KU528628	KU495935	Gąsiorek et al. (2016)
Mesocrista spitsbergensis	KX347532	KX347533	KX347535	Gąsiorek et al. (2016)
Pilatobius recamieri	KX347526	KX347527	KX347529	Gąsiorek et al. (2017)
Pilatobius islandicus	MH682258	MH682257	MH734939	Buda et al. (2018)
Pilatobius glacialis	MW012733	MW012744	MW009692	Zawierucha et al. (2020)
Platicrista aff angustata	MT126762	MT126781	MT107476	Gąsiorek and Michalczyk (2020)

Platicrista horribilis	MT126763	MT126782	MT107477	Gąsiorek and Michalczyk (2020)
Raribius sp.	MT126758	MT126777	MT107472	Gąsiorek and Michalczyk (2020)
**New sequences**				
***Hypsibius* sp.**	**ON898549 (Snow Tardigrada 1)**	**ON927924–ON927925 (Snow Tardigrada 2, 3)**	**ON899873–ON899875 (Snow Tardigrada 1, 2, 3)**	This study
